# Comparison of intravenous sedation using midazolam during dental treatment in elderly patients with/without dementia: a prospective, controlled clinical trial

**DOI:** 10.1038/s41598-021-83122-2

**Published:** 2021-02-11

**Authors:** Yuichi Tatsuno, Yoshinari Morimoto, Megumi Hayashi, Takatoshi Iida

**Affiliations:** Department of Critical Care Medicine and Dentistry, Graduate School of Dentistry, Kanagawa Dental University, 82, Inaoka-cho, Yokosuka, Kanagawa 238-8580 Japan

**Keywords:** Diseases, Medical research

## Abstract

The effects of intravenous sedation with midazolam on the cerebral function of elderly patients with severe dementia are unclear. This study aimed to evaluate its effects on parameters such as brainwaves and cerebral blood flow (CBF) and compare them between elderly individuals with dementia and without cognitive impairment. Ten patients with severe dementia and 10 without cognitive impairment were registered. The bispectral index (BIS) and normalized tissue hemoglobin index (nTHI), which reflects CBF using near-infrared spectroscopy, were measured. Midazolam was administered until a Modified Observer’s Assessment of Alertness/Sedation score of 2 was reached. The chi-squared, Mann–Whitney U, Wilcoxon signed-rank, and Friedman tests and multiple regression analysis were used for comparisons. Whereas a similar decline in BIS values was observed in both groups after midazolam administration (*P* < 0.018), there was a significant decrease by 9% in the nTHI of the dementia-positive group (*P* < 0.013). However, there was no significant difference in the nTHI between the dementia-positive and dementia-negative group according to the multiple regression analysis (*P* = 0.058). In the dementia-negative group, none of the measured values differed from the baseline values. In the dementia-positive group, sedation with midazolam resulted in a 9% decrease in the CBF.

## Introduction

Japan has become a super-aged society in recent years. As the numbers of elderly people requiring long-term care and of those with dementia have risen, there is consequently a rapid increase in the number of such patients undergoing dental treatment. Intravenous sedation is used as a behavior management technique during dental treatment of patients with advanced dementia when they show resistance or refusal to undergo treatment^1^.

Regarding the effects of anesthetics and sedatives on cognitive function, although some clinical studies have not found an increased risk of developing dementia in older individuals or mild cognitive impairment even in those who had undergone general anesthesia in the past^2–6^, there are studies reporting an increase in onset risk associated with their use^7–10^. However, these were retrospective studies based on database and clinical results regarding general anesthesia of patients diagnosed with dementia. These studies have not prospectively assessed the details of cognitive impairments or the effects of anesthetics and sedatives. On the other hand, while basic research on neurons has found that volatile anesthetics (sevoflurane and isoflurane) increase the accumulation of amyloid β proteins and promote tau phosphorylation^11^, intravenous anesthetics, such as propofol, have been reported to inhibit the accumulation of such proteins^12^. However, there is yet to be a consensus for midazolam, as there are contradictory findings reporting that the accumulation of these proteins is inhibited^13^ or enhanced by midazolam^14^.

The sedatives midazolam, propofol, and dexmedetomidine are often used for intravenous sedation during dental treatment^1^. Midazolam is classified as a benzodiazepine sedative. These gamma-aminobutyric acid (GABA) receptor agonists are thought to induce cognitive impairment, such as delirium, postoperatively, and propofol, another GABA receptor agonist, may also be considered to do so^15,16^. Conversely, dexmedetomidine can have cerebral protective effects, such as maintenance of cerebral blood flow (CBF) during hypoxia and focal cerebral ischemia, leading to decreased brain cell damage^17–19^. In many cases, elderly people requiring long-term care and those with dementia show different types of reactions to each sedative compared to healthy individuals with no cognitive impairment. Regarding intravenous sedation with midazolam in the elderly with severe dementia, dose titration is needed to a lower than the usual dose, and respiratory complications (apnea) have occasionally been reported in this population. These conditions were different when other sedatives (propofol or dexmedetomidine) were used^20^.

To maintain a satisfactory perioperative cognitive function, it is generally believed that preserving brain cell activity (brainwaves and CBF) under anesthesia is important. In fact, however, there has been no prospective clinical study to investigate the effects of anesthetics and sedatives on brain cell activity of elderly patients with severe dementia^15,16^. Even when the sedation level is adjusted equally among the use of diverse sedatives, brain function can be affected differently by each drug.

The objective of the present study was to prospectively investigate and compare the effects of intravenous sedation with the most commonly used drug, midazolam, on brainwaves [bispectral index (BIS)] and CBF between elderly individuals with severe dementia and those without cognitive impairment. Elderly patients with severe dementia who were scheduled to undergo behavior management with intravenous sedation for dental treatment were prospectively enrolled. Body composition analysis (frailty assessment) was also performed to evaluate the postoperative cognitive impairment risk due to poor nutrition and sarcopenia.

## Methods

### Study design

This study was conducted in accordance with the Helsinki Declaration. The study protocol was approved by the Institutional Research Board and Ethics Committee of the Kanagawa Dental University (Approval No. 472). The study’s procedures, benefits, safety, and risks were thoroughly explained to all the subjects and/or their guardians and written informed consent was obtained. The study protocol was registered and published in the University Hospital Medical Information Network Center (UMIN Clinical Trails Registry) (ID: UMIN000030338; registered on December 13, 2017).

### Study participants

The cohort comprised patients aged ≥ 60 years who visited the geriatric dental clinic of the Kanagawa Dental University Hospital between January 2018 and December 2019, and were classified as physical status class 1 or 2 of the American Society of Anesthesiologists classification. Of them, patients who were scheduled to undergo dental treatment (including dental extraction) using intravenous sedation with midazolam for particular reasons were selected as potential candidates. The reasons for intravenous sedation were as follows: uncooperative to undergo dental treatment due to advanced dementia, dental phobia, and a severe gagging reflex without cognitive impairment.

Once the schedule of sedation was established, a Mini-Mental State Examination (MMSE) was performed. Subjects with a score of ≥ 24 were considered as “not having a cognitive impairment,” and they were included in the “dementia-negative group”^21^. Conversely, those with an MMSE score of ≤ 23, or those who could not undergo the MMSE due to cognitive decline were considered to have dementia. Of the elderly patients with dementia, those who met the following criteria were considered to have “severe dementia” and were included in the “dementia-positive group”: (1) patients with Alzheimer-type dementia with Functional Assessment Staging of Alzheimer’s disease (FAST) stage 6 or 7 and/or (2) patients classified by the Clinical Dementia Rating (CDR) as “severe.” The MMSE, FAST, and CDR were assessed by medical specialists (MH and TI) other than the attending anesthesiologists (YT and YM). The following patients were excluded: i) those with not severe dementia (MMSE ≤ 23 but with FAST ≤ 5 and/or CDR ≤ 2); (2) who were not able to undergo body composition measurements with the bio-impedance analysis (BIA) method due to diseases of the extremities; and (3) those with allergies or glaucoma who could not take midazolam. All patients with dementia received donepezil and/or memantine hydrochloride.

**Figure 1:**
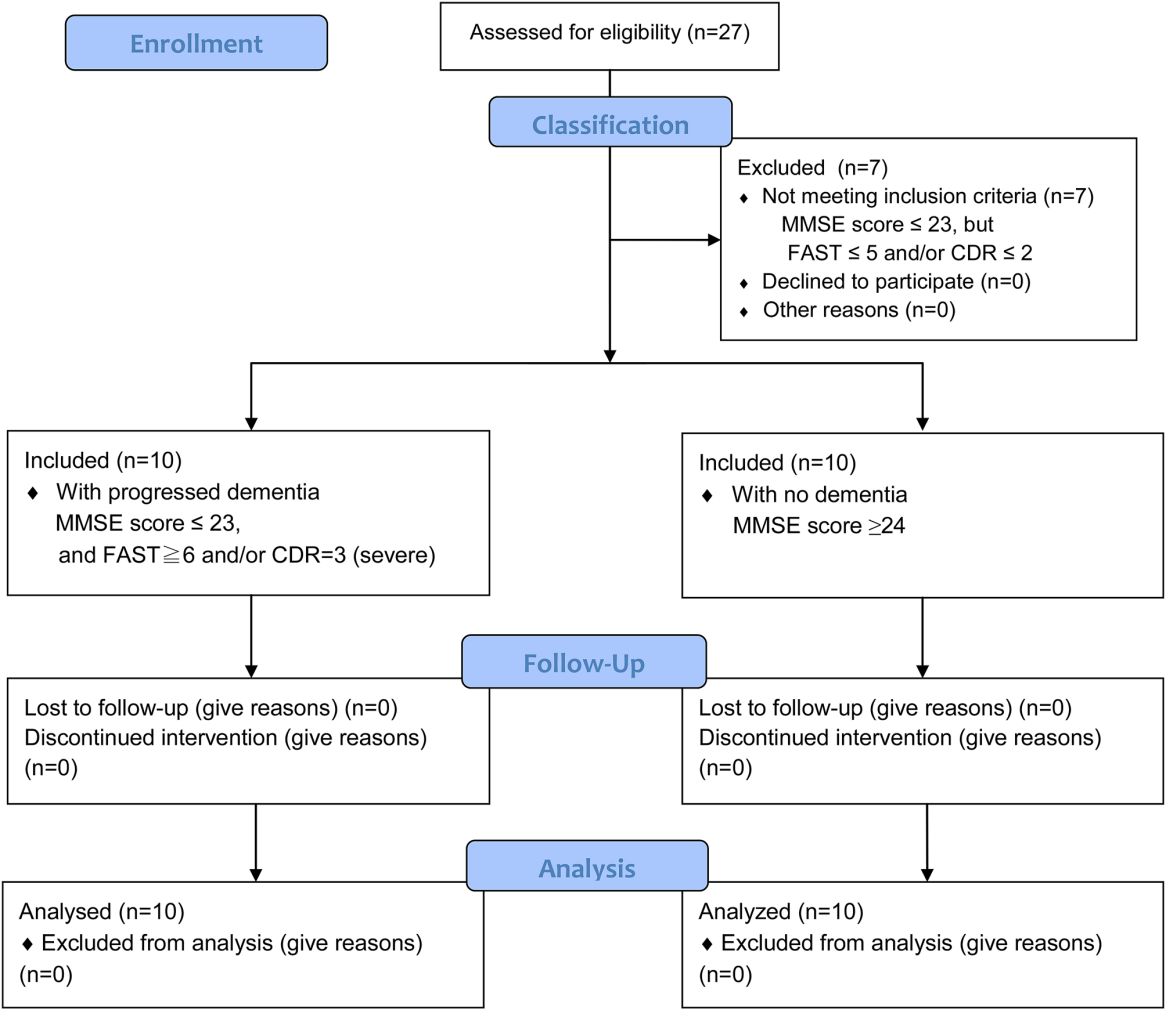
Flow chart showing the participant selection process.

The study procedures were explained to all patients who met the above-mentioned inclusion criteria and/or all of their guardians during the study period. A total of 20 patients who provided consent were included (Fig. 1).

### Recordings

#### Pretreatment fasting and measurement of body composition

Based on the “Intravenous Sedation Guidelines” published by the Japanese Dental Society of Anesthesiology, the participants were asked to refrain from eating and drinking water 8 and 2 h, respectively, before the start of sedation^1^. After arriving at our clinic, the subjects were asked to rest on a bed for 20 min in a quiet room with an approximate temperature of 25 °C. Body composition was measured using the BIA method with a BioScan 920-II (Maltron International Ltd, England). The principle of measurements using the BIA method involves passing a weak, non-invasive electric current through the body and measuring its impedance^22–24^. Muscles contain water and conduct electricity easily. Therefore, the impedance value decreases when muscle mass increases, whereas increases when body fat increases^22–24^. In this study, total body water percentage (/body weight), body fat percentage (/body weight), muscle mass (kg), and protein levels (kg) were measured using the BIA method, and their correlation with the normalized tissue hemoglobin index (nTHI) (values just before the treatment initiation), which reflects CBF of elderly patients with severe dementia, was investigated. All measurements obtained before the treatment were not disclosed to the attending anesthesiologists.

#### Monitoring and sedation method

**Figure 2:**
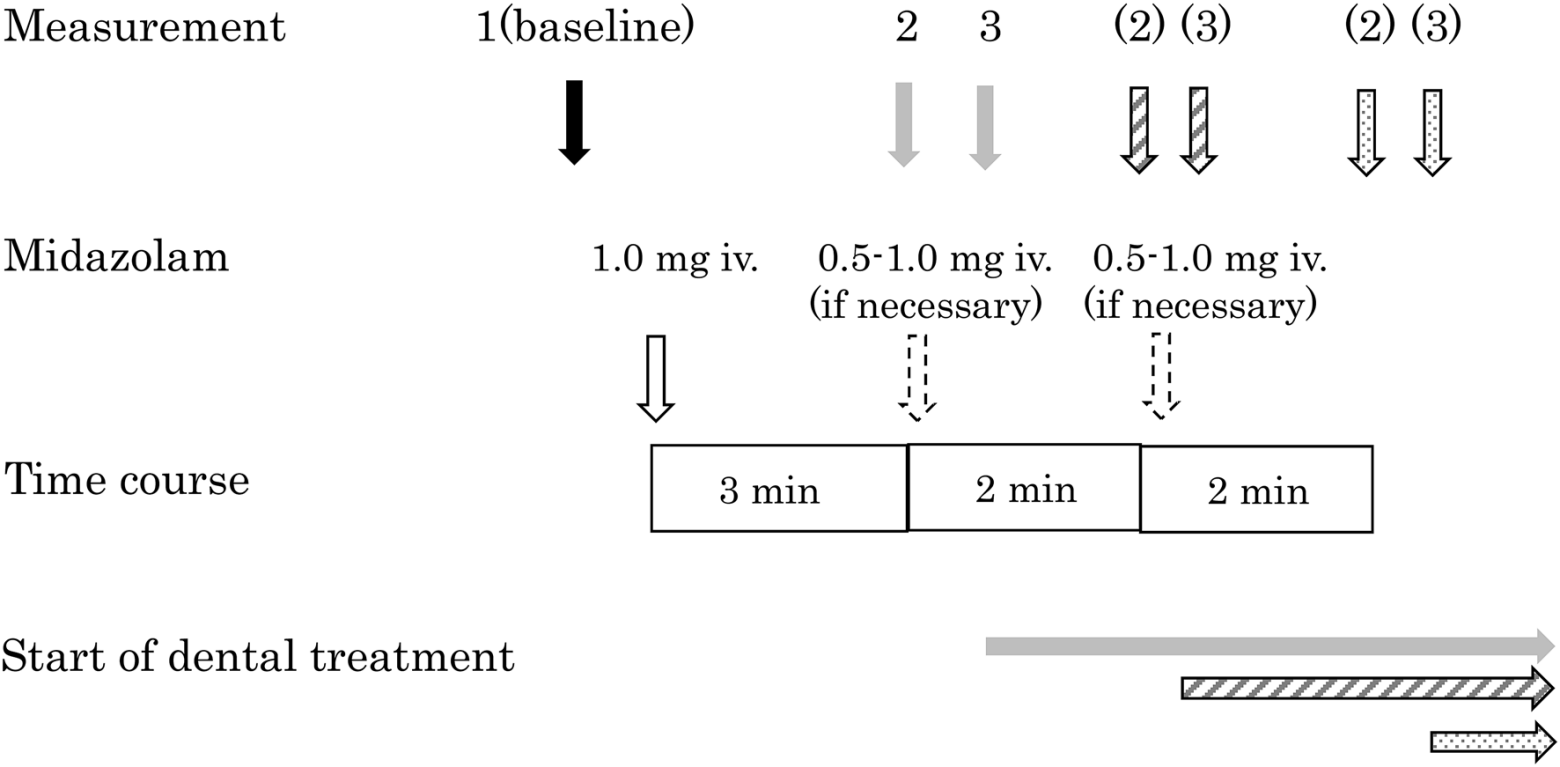
Time course of midazolam administration and evaluation.

Preanesthetic medications were not used. After the abovementioned body composition parameters were measured, the subjects entered the treatment room. Initially, the patients were asked to sit on the treatment table and then lay down on a horizontal position. A non-invasive blood pressure monitor, electrocardiograph, and a percutaneous arterial oxygen saturation (SpO_2_) monitor, as well as BIS sensors and a near-infrared spectroscopy (NIRS) probe (NIRO-200NX; Hamamatsu Photonics CO., Hamamatsu, Japan), were placed on the forehead^25^. A nasal cannula for detecting carbon dioxide (CO_2_) (Smart CapnoLine plus O_2_; Covidien Japan, Tokyo, Japan) was placed, and the end-tidal CO_2_ (etCO_2_) was measured using a Capnostream 20P (Medtronic Covidien Japan Co., Tokyo, Japan). This device uses a nasal cannula to measure exhaled CO_2_, which also allows simultaneous oxygen delivery. The measurements were obtained just before starting the dental treatment. The assistant lightly lifted up the chin of the patient and manually guided the light closure of the patient’s mouth to ensure that CO_2_ measurements were taken when the patient was breathing through the nose only. A peripheral intravenous line was secured using a 22-G intravenous cannula at the dorsum of the right or left hand. The administration of 2 mL/kg/h of Ringer’s acetate solution (Fisio 140: Otsuka Co., Tokyo, Japan; 1% glucose and Ringer’s acetate solution) was started using an infusion pump. The onset of midazolam action is rapid with a peak effect reached within 2–3 min following administration. After intravenous administration, midazolam is rapidly distributed with a distribution half-life of 6–15 min^26^. Based on these pharmacological data, the patients were observed for 3 min after intravenous administration of 1 mg midazolam, and the Modified Observer’s Assessment of Alertness/Sedation (OAA/S) score was assessed to evaluate the sedation level^27^. For this evaluation, the researchers called out the names of all patients (or other words that the patients in the dementia-positive group could respond to; these words were reported by their guardians to the researchers beforehand) loudly and repeatedly followed by prodding or shaking, and their responses were observed to determine whether their sedation level reached an OAA/S score 2 (responds only after mild prodding or shaking). If an OAA/S score of 2 was not achieved at this point, additional administration of 0.5–1 mg of midazolam each time was continued until an OAA/S score of 2 was reached. The OAA/S score was evaluated 2 min after each additional midazolam administration^28^. The treatment was started once an OAA/S score of 2 was achieved (Fig. 2). Researchers other than the attending anesthesiologists evaluated the sedation level.

After sedation, the patients were allowed to breathe room air. Once SpO_2_ reached < 90%, oxygen was delivered at 1 L/min by a nasal cannula. When SpO_2_ < 90% persisted, an additional 1 L/min of oxygen was delivered until SpO_2_ ≥ 90% was reached. Making sure that the attending anesthesiologists (YT and YM) could not see the NIRO measurements and BIS values under sedation, other researchers (MH and TI) recorded the measurements.

#### Measurement parameters and measurement periods

Age, sex, height, body weight, and body mass index (BMI) were collected as patient background characteristics from the medical records. Regarding the length of treatment, sedation time, and dosage of midazolam required to achieve an OAA/S score of 2 were investigated. The parameters measured under sedation were the mean arterial pressure (MAP), heart rate, SpO_2_, etCO_2_, BIS values, and NIRO measurements [left and right cerebral tissue oxygenation index (TOI) and nTHI]^25^.

As for the measurement times during sedation, the TOI and nTHI were assessed at three measurement points: (1) 5 min after the start of measurement, just before midazolam administration (a period of time to stabilize the measurements) (TOI 1, nTHI 1); (2) when the OAA/S score of 2 was reached after midazolam administration (TOI 2, nTHI 2); and (3) just before starting the treatment (TOI 3, nTHI 3). The treatment was started 4–10 min after the first midazolam administration (Fig. 2). Based on the distribution half-life of midazolam that is 6–15 min, an OAA/S score of 2 was considered to remain at measurement point 3^26^.

As the invasiveness differed depending on the dental treatment plan, only measurements up to just before the start of treatment were performed. The MAP, SpO_2_, etCO_2_, and BIS values obtained just before midazolam administration (measurement point 1) were used as the baseline values. For the MAP, SpO_2_, and BIS values, the lowest value measured from the start of midazolam administration and just before the start of treatment (measurement point 3) was recorded, and for etCO_2_, the highest value was recorded.

#### Measurement parameters of the BIA method

Total body water percentage, body fat percentage, muscle mass, and protein levels were measured using the BIA method in the dementia-positive group.

### Statistical analysis

Statistical analysis was performed using SPSS version 16.0 software (SPSS Japan, Tokyo, Japan). Data are presented as medians (interquartile range). The chi-squared test was used to assess sex differences, and the Mann–Whitney U test was used to conduct comparisons of other parameters between the dementia-positive and dementia-negative groups. For the intra-group comparisons, the Wilcoxon signed-rank test was used for the MAP, SpO_2_, BIS, and etCO_2_ values, as they were before-and-after comparisons. The TOI 1, 2, and 3, and the nTHI 1, 2, and 3 comparisons were performed using the Friedman test, and as a post hoc analysis, a Bonferroni-corrected Wilcoxon signed-rank test was performed. Spearman’s rank correlation coefficient was used to determine the correlations between the measurement parameters obtained with the BIA method and nTHI 3 in the dementia-positive group. To examine the independent associations between the nTHI and other variables, multiple regression analysis was performed. Significance was set at *P* < 0.05 (*P* < 0.017 after Bonferroni correction).

The primary endpoint was the nTHI. A preliminary study was conducted as there were no previous studies comparing brain functions between patients with dementia and healthy individuals. Midazolam was administered intravenously (1.0 mg at the first time and 0.5–1.0 mg additionally when necessary) in a total of eight elderly patients (four with and four without dementia; ≥ 60 years old) until an OAA/S score of 2 was reached, and the right nTHI (rnTHI) was measured at that time. The results showed that the rnTHI after midazolam administration in elderly people with and without dementia was on average 0.89 ± 0.09 and 1.06 ± 0.14, respectively. The average standard deviation (0.115) was employed for calculating the required number of cases. According to calculation with the alpha error set at 5% and beta error at 20%, eight individuals per group were required to achieve a power of 80%. Assuming a drop-out rate of 20%, a final sample size of 10 patients in each group (total 20 patients) was required.

## Results

### Patients’ background characteristics

A total of 20 elderly people (5 men, 15 women) with a median age of 74.5 (69.8–78.8) years, median height of 157.5 (156–169) cm, median weight of 48.5 (46.3–59.9) kg, and a median BMI of 19.7 (18.1–24.9) kg/m^2^ participated in the study. The dosage of midazolam was 0.034 (0.023–0.042) mg/kg. The dementia-positive group consisted of eight patients with Alzheimer-type dementia, one patient with Lewy body dementia, and one patient with frontotemporal dementia. The FAST stages of the eight patients with Alzheimer-type dementia were as follows: two patients in stage 6e, two patients in stage 7a, and four patients in stage 7b. The CDR for all these patients was “severe.” The dementia-negative group consisted of eight patients with dental phobia, and two with a severe gagging reflex.

### Comparisons of patients’ background characteristics

There were no differences in age, weight, treatment length, and sedation time between the dementia-positive and dementia-negative groups. While the dementia-positive group comprised women only (*P* = 0.023), and there was a significant difference observed in height (*P* = 0.002) and BMI (*P* = 0.013), these differences were not considered to be clinically significant. Furthermore, the dosage of midazolam needed to achieve an OAA/S score of 2 was 0.027 mg/kg (median) in the dementia-positive group and 0.035 mg/kg (median) in the dementia-negative group, suggesting a significantly lower dose required in the dementia-positive group (*P* = 0.052) (Table 1). The data of body composition analysis in the dementia-positive group are presented in Table 1.

**Table 1:** Characteristics of the participants.

	Patients with dementia	Patients without dementia	*P* value	MW-U
Age (years)	72 (68.8–74.3)	78 (72.8–80.8)	0.089	27.0
Sex (male/female)	0/10	5/5	0.023	20.0
Height (cm)	156.5 (152.4–157.5)	169.0 (160.9–171)	0.002	9.0
Weight (kg)	48.5 (47–64.7)	52.5 (45.9–58.5)	0.247	34.0
BMI	22.5 (19.7–26.1)	18.1 (16.6–20.9)	0.013	15.0
Treatment time (min)	42.5 (36.3–62.3)	60.5 (43.5–68)	0.165	31.0
Sedation time (min)	65.5 (51.5–88.3)	83 (64.8–87.5)	0.247	34.5
Initial dose of midazolam (mg/kg)	0.027 (0.017–0.036)	0.035 (0.033–0.057)	0.052	24.0
Body composition analysis				
Total body water (%)	60.9 (54.6–66.6)			
Fat volume (%)	26.4 (17.4–30.3)			
Muscle volume (%)	22.5 (19.5–26.7)			
Protein volume (%)	11.2 (9.7–13.1)			

*BMI* body mass index, *MW-U* Mann–Whitney U value.

Eleven patients (seven in the dementia-positive and four in the dementia-negative group) reached an OAA/S score of 2 following the first administration of midazolam, eight patients (three and five in the dementia-positive and dementia-negative groups, respectively) after the second administration, and one patient (dementia-negative group) after the third administration. An OAA/S score of 2 was remained for all participants when dental treatments were initiated.

**Figure 3:**
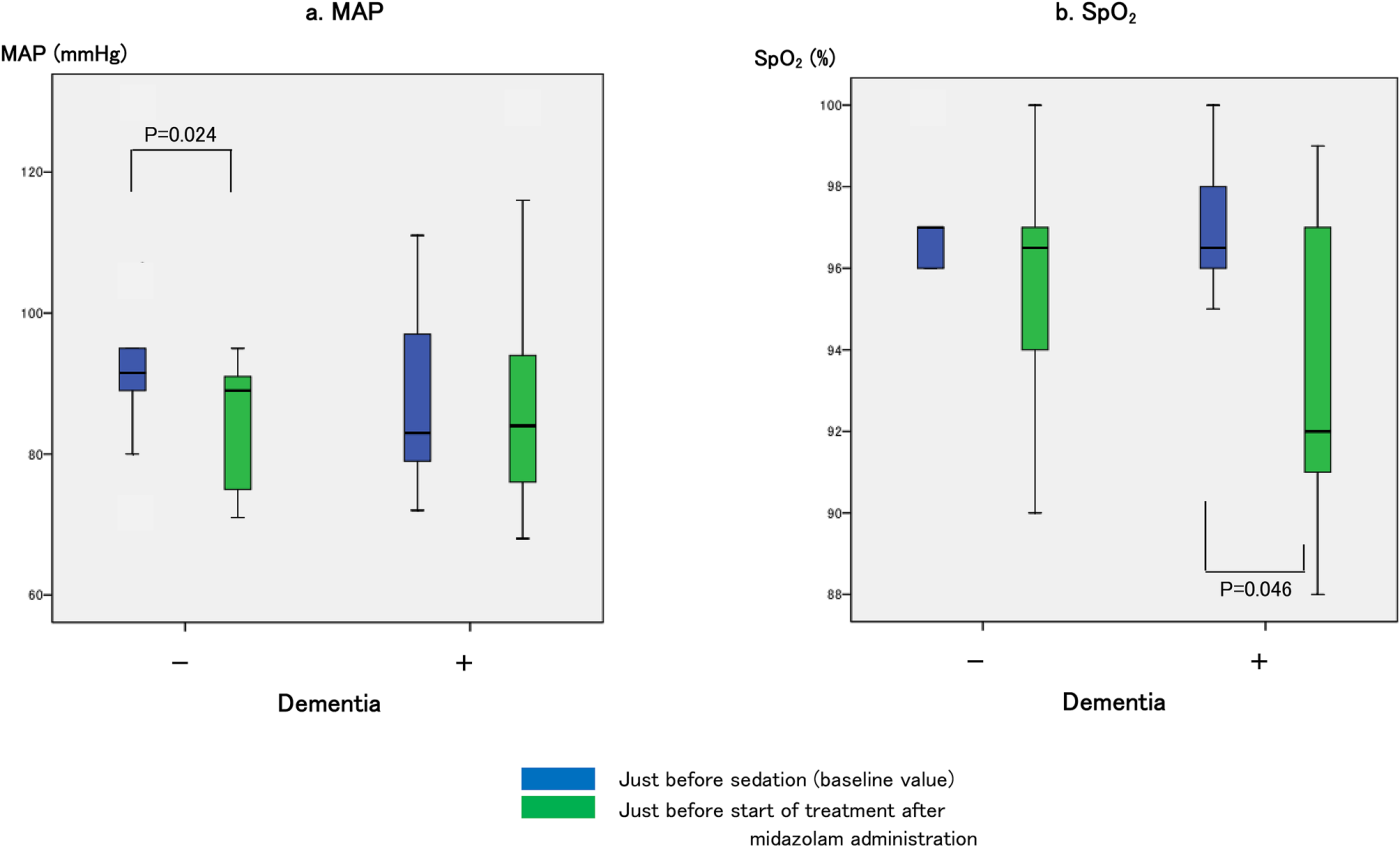
Changes in the mean arterial pressure (MAP) and percutaneous arterial oxygen saturation (SpO_2_). (**a**) indicates the MAP values: 91.5 (86.8–97.5) mmHg just before sedation and 89 (74.5–91) mmHg just before the start of treatment after midazolam administration (*P* = 0.024) in the dementia-negative group; 83 (78.5–99.3) mmHg just before sedation and 84 (74.5–99.5) mmHg just before the start of treatment after midazolam administration in the dementia-positive group. (**b**) indicates the SpO_2_ values: 97% (96–97) just before sedation and 96.5% (93.8–97.3) just before the start of treatment after midazolam administration in the dementia-negative group; 96.5% (96–98) just before sedation and 92% (90.5–97.5) just before the start of treatment after midazolam administration (*P* = 0.046) in the dementia-positive group.

**Figure 4:**
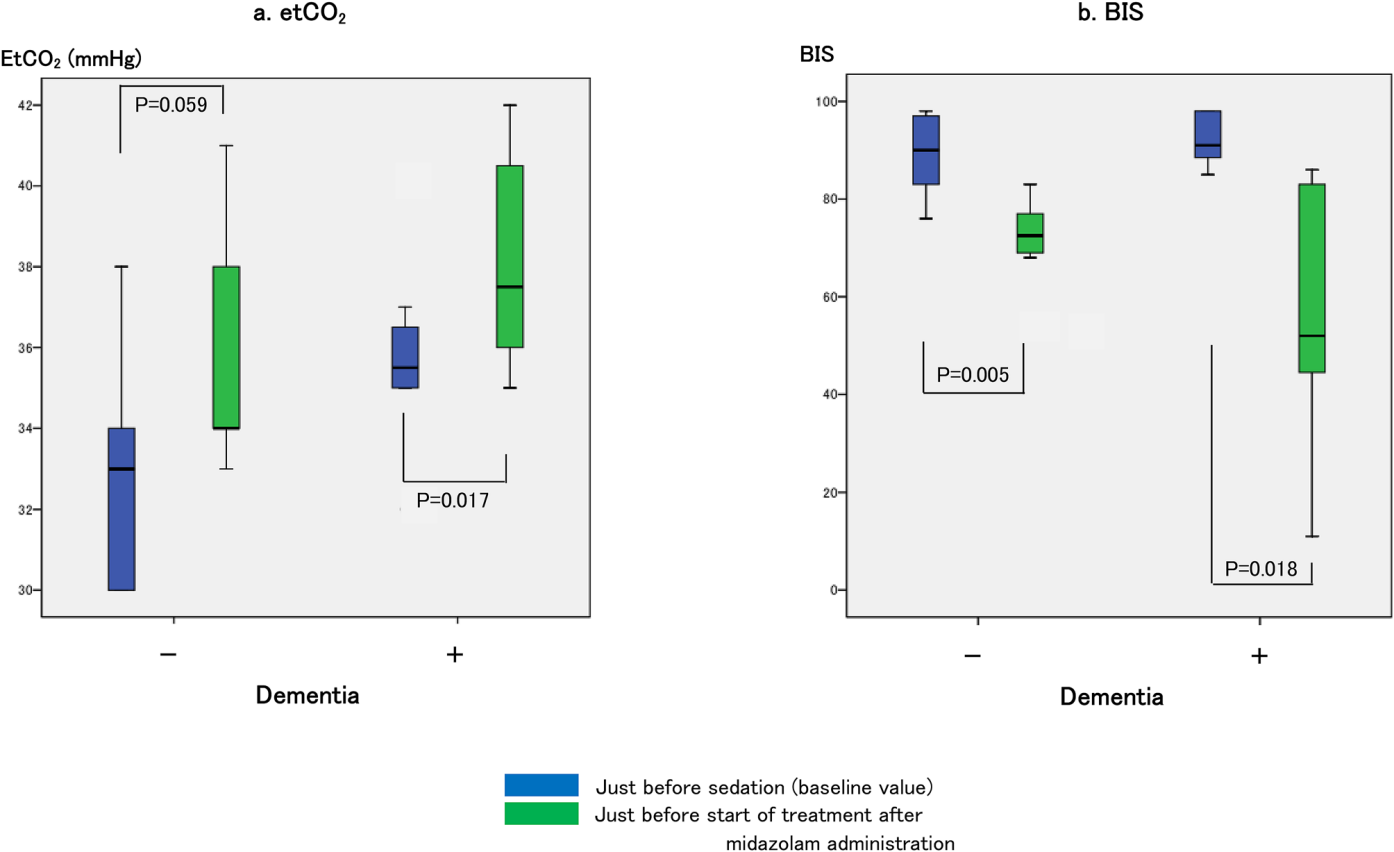
Changes in the end-tidal CO_2_ (etCO_2_) and bispectral index (BIS) values. (**a**) indicates the etCO_2_ values: 33 (30–36) mmHg just before sedation and 34 (33.5–39.5) mmHg just before the start of treatment after midazolam administration in the dementia-negative group; 35.5 (35–36.8) mmHg just before sedation and 37.5 (36–40.8) mmHg just before the start of treatment after midazolam administration in the dementia-positive group (*P* = 0.017). (**b**) indicates the BIS values: 90 (82.8–97) just before sedation and 72.5 (68.8–77.8) just before the start of treatment after midazolam administration (*P* = 0.005) in the dementia-negative group; 91 (87–98) just before sedation and 52 (37–85) just before the start of treatment after midazolam administration (*P* = 0.018) in the dementia-positive group.

There were no differences in the MAP, SpO_2_, etCO_2_ and BIS values between the two groups in terms of the baseline values and those before the start of treatment (Figs. 3 and 4). However, on intra-group comparison, the BIS values after midazolam administration in both groups were significantly lower (Fig. 4). However, after midazolam administration, the level of etCO_2_ increased (median 35.5–37.5 mmHg) and of SpO_2_ decreased (median 96.5–92.0%) in the dementia-positive group, and the MAP decreased (median 91.5–89 mmHg) in the dementia-negative group (Figs. 3 and 4).

### Results of the NIRS measurement parameters

#### Changes in the nTHI

##### Comparisons between the dementia-positive and dementia-negative groups

**Figure 5:**
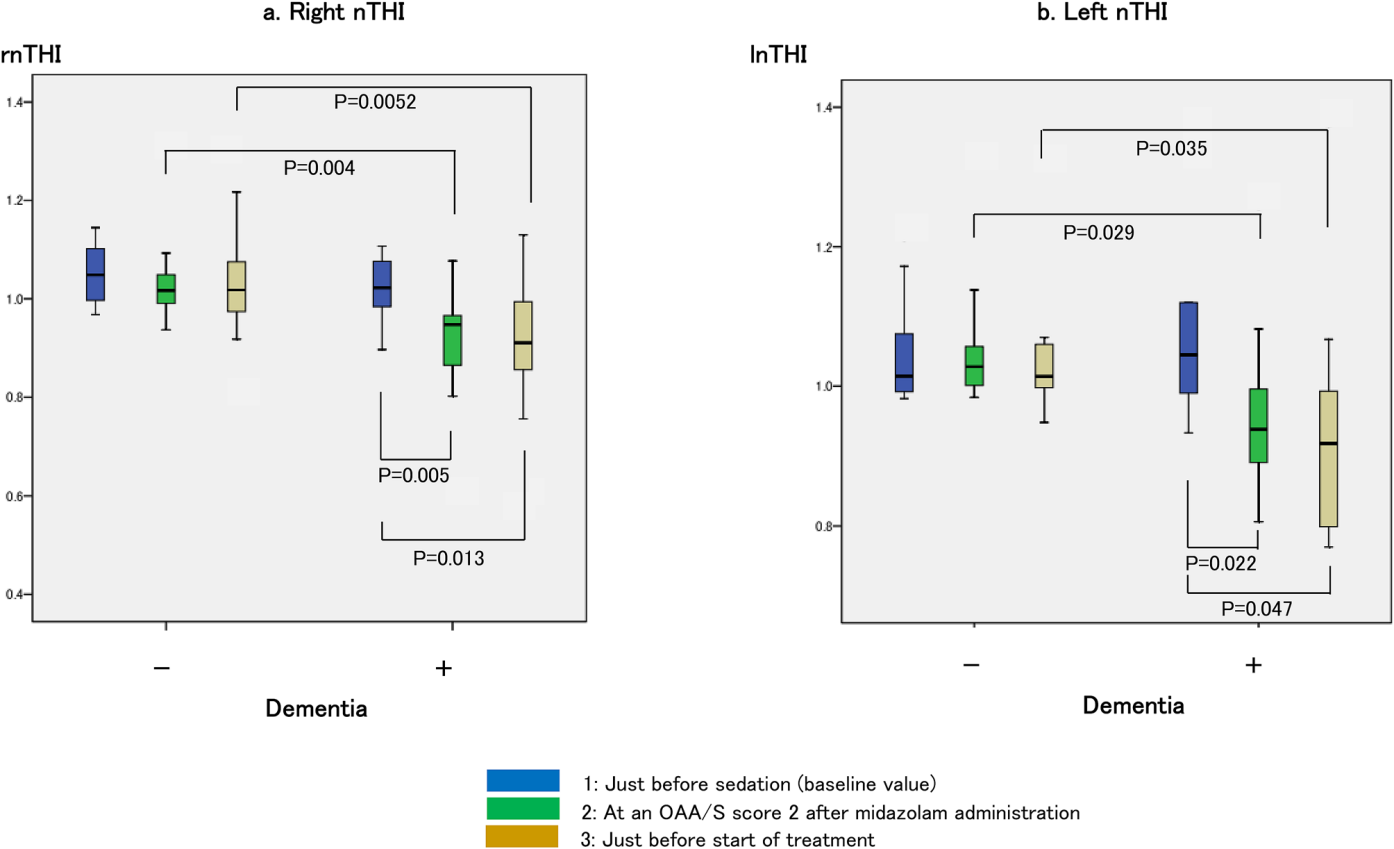
Changes in the normalized tissue hemoglobin index (nTHI). (**a**) indicates the right nTHI values: 1.05 (0.99–1.11) just before sedation (baseline value), 1.02 (0.99–1.06) when OAA/S score of 2 was achieved after midazolam administration, and 1.02 (0.96–1.09) just before the start of treatment in the dementia-negative group; 1.02 (0.98–1.08) just before sedation (baseline value), 0.95 (0.85–0.97) when OAA/S score of 2 was achieved after midazolam administration, and 0.91 (0.83–0.99) just before the start of treatment in the dementia-positive group. (**b**) indicates the left nTHI values: 1.02 (0.99–1.10) just before sedation (baseline value), 1.03 (1.00–1.08) when OAA/S score of 2 was achieved after midazolam administration, and 1.01 (1.00–1.06) just before the start of treatment in the dementia-negative group; 1.05 (0.98–1.17) just before sedation (baseline value), 0.94 (0.87–1.02) when OAA/S score of 2 was achieved after midazolam administration, and 0.92 (0.79–1.01) just before the start of treatment in the dementia-positive group. The right nTHI values are significantly different among the three measurement points in the dementia group (*P* = 0.008, χ^2^ = 9.600), and post hoc analysis was performed (indicated in the figure). The left nTHI values showed no significant difference among the three measurement points in the dementia group (*P* = 0.097, χ^2^ = 4.667), but the left nTHI values suggest a decreasing trend after midazolam administration. Each comparison of data (measurement point 2 or 3) between those with and without dementia is close to significant.

The measurement of the nTHI at the reference point [nTHI 1 on the right (rnTHI 1) and nTHI 1 on the left (lnTHI 1)] showed the baseline value (1) in both groups. However, for the rnTHI measurements, rnTHI 2 and rnTHI 3 of the dementia-positive group decreased compared to the dementia-negative group (*P* = 0.004 and *P* = 0.0052, respectively). For the lnTHI measurements, lnTHI 2 and lnTHI 3 of the dementia-positive group decreased compared to the dementia-negative group (*P* = 0.029 and *P* = 0.035, respectively) (Fig. 5).

##### Comparisons within groups

In the dementia-positive group, significant changes were observed in the rnTHI values. When reviewed individually, rnTHI 2 and rnTHI 3 decreased by 5% and 9%, respectively, compared to rnTHI 1 (baseline value 1), and each decreased significantly (*P* = 0.008, χ^2^ = 9.600; post hoc analyses were *P* = 0.005 and *P* = 0.013, respectively). The lnTHI decreased by approximately 8%; however, there was no significant difference (*P* = 0.097, χ^2^ = 4.667). All measurements on both sides in the dementia-negative group showed the baseline value (1), and no significant change was observed (Fig. 5).

#### Changes in the TOI

**Figure 6:**
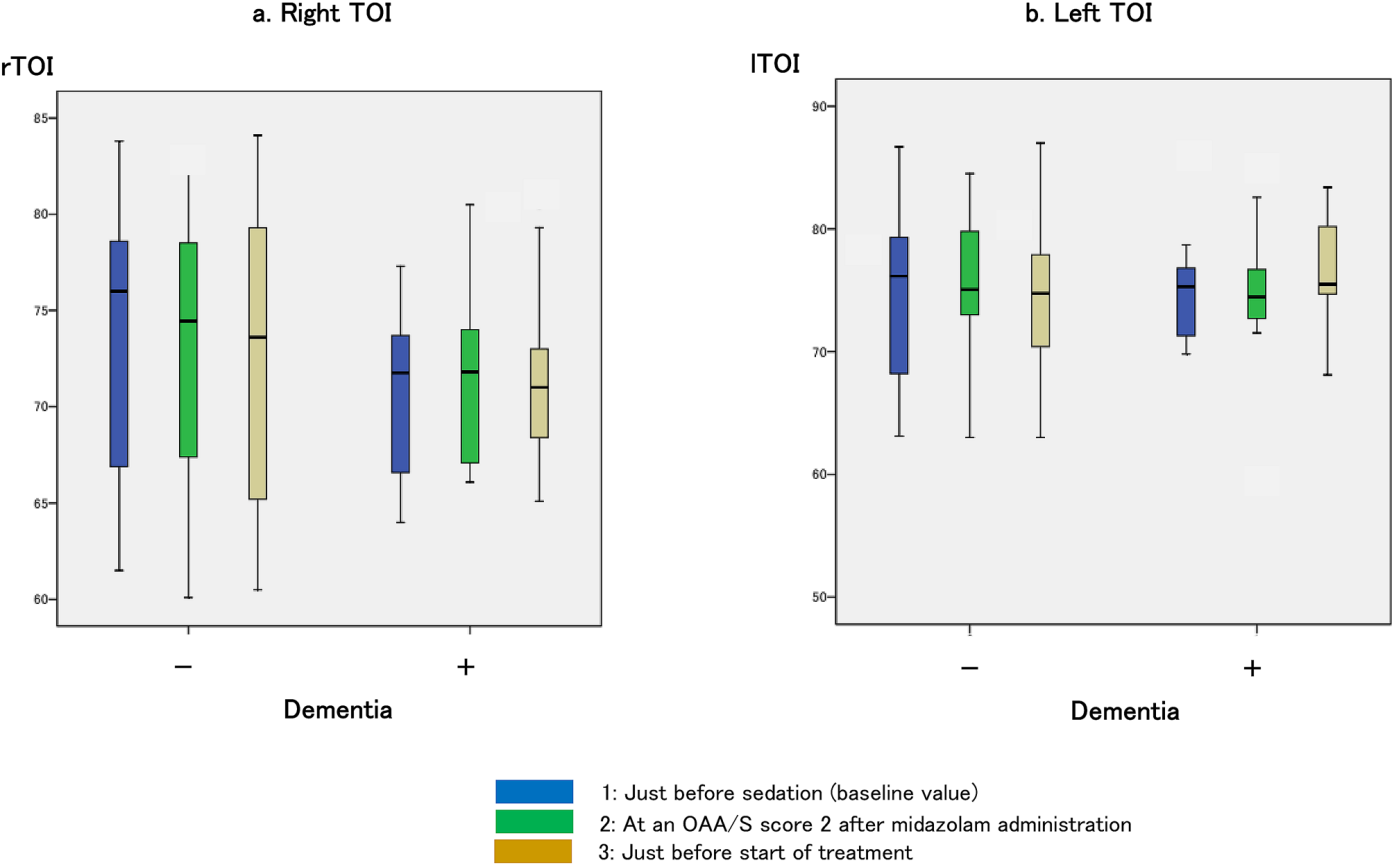
Changes in the tissue oxygenation index (TOI). (**a**) indicates the right TOI values: 76.0% (66.4–79.7) just before sedation (baseline value), 74.5% (66.9–79.5) when OAA/S score of 2 was achieved after midazolam administration, and 73.6% (65–79.6) just before the start of treatment in the dementia-negative group; 71.8% (66.4–74.4) just before sedation (baseline value), 71.8% (66.9–74.5) when OAA/S score of 2 was achieved after midazolam administration, and 71.0% (67.8–74.6) just before the start of treatment in the dementia-positive group. (**b**) indicates the left TOI values: 76.2% (67.1–80.9) just before sedation (baseline value), 75.1% (70.7–80.9) when OAA/S score of 2 was achieved after midazolam administration, and 74.8% (68.6–79.5) just before the start of treatment in the dementia-negative group; 75.3% (71.2–77.3) just before sedation (baseline value), 74.5% (72.4–78.2) when OAA/S score of 2 was achieved after midazolam administration, and 75.5% (74.4–80.6) just before the start of treatment in the dementia-positive group.

There was no difference in TOI changes between the dementia-positive and dementia-negative groups at each measurement point. There were also no significant changes within each group (Fig. 6).

#### The independent associations between the nTHI and the other variables

In the multiple regression analysis, the values of the nTHI (rnTHI 2, rnTHI 3, lnTHI 2, and lnTHI 3) were used as a dependent variables, whereas the independent variables included the presence of dementia; the lowest values of SpO_2_, MAP, and BIS; and the highest value of etCO_2_. A decrease was found in the nTHI in patients with dementia, but there was no significance in the nTHI between the dementia-positive and dementia-negative groups according to the multiple regression analysis (rnTHI 2: β =  − 0.067, *P* = 0.058). The highest value of etCO_2_ (β =  − 0.029, *P* = 0.049) was statistically associated with the decrease in lnTHI 2 (Table 2).

**Table 2:** The independent associations between the nTHI and other variables.

Independent variable	Dependent variable	β	r	*P*
The presence of dementia	rnTHI 2	− 0.067	0.560	0.058
	R^2^ = 0.314	adj.R^2^ = 0.245		(*P* = 0.058)
The value of etCO_2_	lnTHI 2	− 0.029	0.578	0.049
	R^2^ = 0.334	adj.R^2^ = 0.268		(*P* = 0.049)

Definition of independent variable: presence of dementia (1), and no presence of dementia (0).

*rnTHI2* right normalized tissue hemoglobin index, *lnTHI* left normalized tissue hemoglobin index.

#### Correlation between the nTHI and body composition measurement parameters

In the dementia-positive group, lnTHI 3 value was found to correlate with both muscle mass (%) and protein levels (%) (*P* = 0.048). However, rnTHI 3 value did not correlate with any of the measured body composition parameters (Table 3).

**Table 3:** Correlation between rnTHI 3 or lnTHI 3 and items in body composition analysis in patients with dementia. Abbreviations: rnTHI: right normalized tissue hemoglobin index, lnTHI: left normalized tissue hemoglobin index, BMI: body mass index.

	rnTHI		lnTHI	
	r	*P* value	r	*P* value
Body weight	− 0.105	0.772	− 0.322	0.364
BMI	− 0.374	0.287	0.184	0.611
Total body water	0.216	0.549	− 0.253	0.480
Fat volume	− 0.327	0.356	− 0.241	0.503
Muscle volume	− 0.241	0.503	− 0.636	0.048
Protein volume	− 0.241	0.503	− 0.636	0.048

#### Changes in cognitive function

The cognitive function of the patients in the dementia-positive group was assessed using the FAST and CDR when the patients were discharged after sedation. None of the patients showed cognitive decline.

## Discussion

This study demonstrated that a lower dosage of midazolam is needed to reach a sedation level of OAA/S score 2 in elderly patients with severe dementia compared to elderly individuals with no cognitive decline. Furthermore, when midazolam was used to bring the sedation level to an OAA/S score of 2, the nTHI (that reflects the CBF) in elderly patients with no cognitive decline remained at a steady level, whereas the nTHI of elderly patients with severe dementia decreased by 9%. Multiple regression analysis suggested that the presence of dementia and the highest value of etCO_2_ were likely to correlate with the decrease in the nTHI. However, the decrease in the BIS value was comparable to that observed in elderly individuals with no cognitive decline.

The NIRS method used emits near-infrared light at three different wavelengths (775, 810, and 850 nm) into the brain tissue, and scattered reflected light from the three different near-infrared light sources irradiated into the tissue is measured at multiple locations of varying distances. Oxygenated hemoglobin, deoxygenated hemoglobin, total hemoglobin concentration, and the TOI were assessed by measuring the light attenuation rate^25,29^. The TOI is defined as the ratio of oxygenated to total hemoglobin concentrations^30^, and the nTHI as the ratio of the hemoglobin concentration at the measurement point to the total hemoglobin concentration at the start of measurement. The nTHI is known to sensitively reflect the CBF at the location on the forehead where the probes are placed^31^. Factors that affect the measurements are anemia, extracranial blood flow, and body position^25^. The present study avoided these obstacles by: (1) ensuring that there was no anemia with a level of hemoglobin < 10 g/dL; (ii) paying attention to avoiding areas with cutaneous veins when placing the probes on forehead; and (iii) taking measurements after the participants rested for 5 min in an horizontal position.

There is no agreement regarding changes in the CBF with the use of midazolam. Some studies have observed a dose-dependent decrease in the cerebral metabolic rate of oxygen (CMRO_2_)_,_ a decrease in CBF, and an increase in cerebral venous resistance (CVR) when midazolam was administered to dogs and humans^32–34^. However, as midazolam was used as an induction agent for general anesthesia in these studies, the dosages were extremely high (0.15–10.0 mg/kg) compared to those used for intravenous sedation in the present study. However, as a decline in the MAP and an increase in PaCO_2_ were observed in these studies, the possibility that these could have affected the changes in the CBF and CVR cannot be excluded. On the other hand, Knudsen et al. conducted a study in which 0.3 mg/kg of midazolam and 4 µg/kg/h of fentanyl were administered to induce anesthesia in human subjects, and anesthesia was maintained with 67% nitrous oxide, 4 µg/kg/h of fentanyl, and 0.125–0.68 mg/kg/h of midazolam. No significant changes in the MAP or PaCO_2_ were observed in this study, and there were no changes in the CBF or CMRO_2_^35^. Reinsel et al. conducted a study using positron emission tomography and reported an approximate 12% reduction in the CBF with the administration of 7.5 mg or 9.5 mg of midazolam when adjusted for the effect of increased PaCO_2_^36^. As resulting from the above, the midazolam dosages in many of the previous studies were substantially higher compared to the dosage used for sedation in the present study, and additional drugs were also used to achieve general anesthesia. While some studies have reported that increased midazolam dosage results in decreased CBF, CMRO_2_, and brainwave activity, other studies have shown that the levels of these parameters are maintained^32–36^.

An adequate dose for intravenous sedation is considered to be 0.05–0.075 mg/kg of midazolam when the sedation level corresponds to an OAA/S score of 2^37^. The midazolam required for sedation is decreased approximately to 75% in elderly adults (calculated as 0.038–0.056 mg/kg)^38^. In the present study, the midazolam dosage in elderly patients with no cognitive decline was 0.035 mg/kg (median value), which was comparable to the current dosage recommended as a standard midazolam dosage for sedation to achieve an OAA/S score of 2^37,38^. Therefore, it was suggested that, at the current regular level of midazolam dosages to achieve an OAA/S score of 2, the CBF is maintained in elderly patients with no cognitive decline. Typically, as the CBF is maintained at a steady level at a MAP range of 70–150 mmHg (autoregulation of CBF), and changes on PaO_2_ from 60 (equivalent to SpO_2_ 90%) to 300 mmHg have little influence on CBF, the response (CBF is maintained even with midazolam administration) of the elderly patients with no cognitive decline is physiologically understandable^39^. Meanwhile, there was an 9% reduction in the CBF in elderly patients with severe dementia with a midazolam dosage lower (median 0.027 mg/kg) than that used for elderly patients with no cognitive decline to achieve an OAA/S score of 2. This CBF reduction in the dementia-positive group was also assessed by multiple regression analysis that suggested the presence of dementia as an associated factor. Many studies suggest that the CBF reduction with midazolam is the result of the suppression of brain tissue metabolism^39^. Elderly patients with severe dementia might have had a weakened CBF autoregulation mechanism or already decreased brain tissue metabolism.

The CBF increases 1–2 mL/100 g/min for each 1 mmHg increase in PaCO_2_ around normal PaCO_2_ values^39^. In this study, the multiple regression analysis proposed that lnTHI 2 negatively correlates with the highest value of etCO_2_, which seems to contradict the physiological findings. This means that lnTHI 2 (that reflects the CBF at an OAA/S score of 2) decreased even though that offset increase in the CBF caused by etCO_2_ level elevation. Based on this fact, sedation with midazolam at an OAA/S score of 2 is likely to reinforce CBF reduction in patients with severe dementia even when etCO_2_ level increases by 2 mmHg (median value). However, as the decrease in left nTHI 1–3 suggested no statistically significant differences, the data indicating that lnTHI 2 negatively correlates with the highest value of etCO_2_ might be underestimated.

This study showed that, whereas the left and right nTHI exhibited similar reductions in patients with dementia, there was no significant difference in the lnTHI (*P* = 0.097). As one of the reasons may have been the small distribution range of the lnTHI 2 data, the results appear to be reasonable. In electroconvulsive therapy for schizophrenia, a study has reported a significant increase in the lnTHI compared to the rnTHI, and the possibility that there may be a bilateral difference in CBF fluctuation depending on the inherent brain disorder cannot be ruled out^31^.

As the participants of the present study could spontaneously breath without tracheal intubation, etCO_2_ measurements were taken using a special nasal cannula for measuring CO_2_, and the relative values were compared. The measurements were performed before starting the dental treatment. The patient’s chin was lifted up lightly to secure the airway, and the mouth was lightly closed manually to measure CO_2_ respiration from the nose only. Therefore, the changes in relative values appear to be reasonable.

The brainwaves, indicated by the BIS values, showed a similar reduction in both the elderly patients with dementia and those with no cognitive decline, and there was no difference. The reason why the participants retained certain physical abilities such as walking despite neurodegeneration from dementia was that some parts of the brain were compensating for brainwave suppression in other parts. While there was a decrease in the CBF with one-time administration of midazolam in patients with severe dementia at FAST stages 6d to 7b, the fact that dementia grades (FAST and CDR) of the patients did not decrease after sedation, and as there was no difference in brainwave activity suppression compared to elderly individuals with no cognitive decline, we concluded that there was no decline in cognitive function. However, as dental treatments may involve multiple visits, and the effects of midazolam over numerous administrations remain elusive, careful attention is needed.

Herein, there were found no changes in the TOI, both in the dementia-positive and the dementia-negative groups. While the TOI does not change as rapidly as arterial oxygen partial pressure and SpO_2_, studies have also reported an increase in the TOI with oxygen administration^40^. The TOI may not have changed in the present study as oxygen was administered at 1 L/min to maintain oxygenation once SpO_2_ < 90% was reached.

In this study, there was found a correlation between reduced CBF only in the left side and muscle mass and protein levels (body composition parameters) in elderly patients with dementia. A decline in physical function due to frailty and sarcopenia associated with aging has been reported in many studies^41^. A firm difference may not have been observed in this study due to the limited sample size; thus, further investigation is needed.

However, this study has some limitations. The probes for measuring the brainwaves (BIS) and the NIRO200NX had to be placed on the forehead, and although the brainwaves and CBF of the forehead could be measured, the conditions at other locations in the brain were not evaluated. In studies with volunteer subjects, measurements at various locations of the brain can be performed. However, during dental treatment, the only location that provides stable measurements is the forehead, and that is a limitation. Although this study was satisfied with the statistical sample size calculation, it comprised a small sample size that is another limitation. In the future, a large-scale study should evaluate cerebral and cognitive function during and after sedation with midazolam. Herein, the sedation level was evaluated using the OAA/S score as a subjective method, which is a limitation. The sedation levels are mainly evaluated by subjective assessment such as the OAA/S score, Sedation-Agitation Scale, and Richmond Agitation-Sedation Scale etc. Although the BIS value calculated based on the brain wave pattern seems an objective assessment, it has been demonstrated that the BIS value does not necessarily correlate with the sedation level accurately^42,43^. When another objective assessment method develops, more accurate evaluation of the sedation level can be achieved. Moreover, each category of the dementia grade classifications (FAST and CDR) has a certain range of symptom findings. If cognitive decline is very subtle, and the decline falls within same grading scale, the results could be classified into the same grade, pointing to a weak detection capability.

In conclusion, the present study demonstrated that when midazolam was used to bring the sedation level to an OAA/S score of 2, the nTHI (that reflects the CBF) in elderly patients with no cognitive decline remained at a steady level, while the nTHI of elderly patients with severe dementia decreased by 9%. However, the decrease in the BIS value was comparable to that observed in elderly patients with no cognitive decline. Based on these results, further comparisons among midazolam and other sedatives in the elderly with severe dementia are warranted in the future.
